# Supplementary material for: In vitro modeling of the human dopaminergic system using spatially arranged ventral midbrain–striatum–cortex assembloids
Source: Nat Methods. 2023 Dec 5;20(12):2034–47. doi: 10.1038/s41592-023-02080-x (PMC10703680; doi:10.1038/s41592-023-02080-x)
Supplement: Supplementary file 1 — Supplementary Tables 1–3 [file 41592_2023_2080_MOESM1_ESM.pdf]

nature methods

Article https://doi.org/10.1038/s41592-023-02080-x

In vitro modeling of the human dopaminergic system using spatially arranged ventral midbrain–striatum–cortex assembloids

In the format provided by the authors and unedited

Supplementary Table S1- qPCR primers

Gene	Forward primer	Reverse Primer
**ACTB**	CCTTGCACATGCCGGAG	GCACAGAGCCTCGCCTT
**GAPDH**	TTGAGGTCAATGAAGGGGTC	GAAGGTGAAGGTCGGAGTCA
**SHH**	CCAATTACAACCCCGACATC	AGTTTCACTCCTGGCCACTG
**EN1**	CGTGGCTTACTCCCCATTTA	TCTCGCTGTCTCTCCCTCTC
**FOXA2**	CCGTTCTCCATCAACAACCT	GGGGTAGTGCATCACCTGTT
**CORIN**	CATATCTCCATCGCCTCAGTTG	GGCAGGAGTCCATGACTGT
**NURR1**	CAGGCGTTTTCGAGGAAAT	GAGACGCGGAGAACTCCTAA
**OTX2**	ACAAGTGGCCAATTCACTCC	GAGGTGGACAAGGGATCTGA
**LMX1A**	CGCATCGTTTCTTCTCCTCT	CAGACAGACTTGGGGCTCAC
**LMX1B**	CTTAACCAGCCTCAGCGACT	TCAGGAGGCGAAGTAGGAAC
**TH**	CGGGCTTCTCGGACCAGGTGTA	CTCCTCGGCGGTGTACTCCACA
**TBR1**	TCGTCCCCGCTCAAGAGCGA	CCTTGGCGCAGTTCTTCTCGCA
**PAX6**	TGGTATTCTCTCCCCCTCCT	TAAGGATGTTGAACGGGCAG
**FOXG1**	TGGCCCATGTCGCCCTTCCT	GCCGACGTGGTGCCGTTGTA
**FOXA2 (SAG dose-curve)**	GCTGGTCGTTTGTTGTGGC	AGGCCGGCGTTCATGTTG

Supplementary Table S2- Primary Antibodies

Species	Antigen	Producer	Cat#	Dilution used in 2D*
Mouse	ALDH1A1 (Clone B-5)	Santa Cruz Biotech	sc-374149	1:50
Rabbit	Calbindin	Aves lab/Swant	CD38a D-28K	1:1000
Rat	CTIP2/ BCL11b	Abcam	ab18465	1:300
Rabbit	DARPP32 (Clone EP720Y)	Abcam	ab40801	1:100
Goat	DARPP32	R&D systems	AF6259	1:100
Rat	DAT (Clone DAT-NT)	Millipore	MAB369	1:1000
Goat	DLX2	Santa Cruz Biotech	sc-18140	1:100
Sheep	DLX5	R&D Systems	AF6710	1:100
Rabbit	DRD1	Abcam	ab20066	1:400
Rabbit	DRD2	Millipore	AB5084P	1:100
Rabbit	DsRed/tdtomato/Crimson	Clontech	632496	1:250
Rabbit	EN2	LSBio	LS-B9057-200	1:100
Goat	FOXA2	R&D Systems	AF2400	1:300
Rabbit	FoxG1	Abcam	ab18259	1:200
Rabbit	FOXP1	Abcam	AB16645	1:200
Rabbit	GABA	Sigma-Aldrich	a2052	1:200
Mouse	GAD67 (Clone 1G10.2)	Millipore	MAB5406	1:200
Chicken	GFP	Aves Labs	GFP-1020	1:500
Rabbit	GIRK2 (Kir3.2)	Alomone Labs	APC-006	1:400
Rabbit	GSX2/GSH	Millipore	ABN162	1:100
Rabbit	ISL-1	Abcam	AB20670	1:100
Rabbit	LMX1a	Sigma-Aldrich	AB10533	1:100
Rabbit	MASH1/ASCL1	Abcam	AB74065	1:100
Rabbit	Nkx2.1/Thyroid (TTF1) (Clone EPR5955(2))	Epitomics	6594-1	1:1000
Goat	OTX2	R&D Systems	AF1979	1:100
Mouse	PAX6 (Clone AD2.38)	Abcam	ab78545	1:100
Sheep	PAX6	R&D Systems	AF8150	1:200 of 100 µl reconstitute
Rabbit	RFP/tdtomato/Crimson	Abcam	ab62341	1:100
Rabbit	SOX2	Abcam	ab97959	1:600
Goat	SOX2	R&D Systems	AF2018	1:100
Rabbit	SOX6	Abcam	ab30455	1:500
Rabbit	TBR1	Abcam	ab31940	1:300
Rabbit	TH	Abcam	ab112	1:300
Sheep	TH	Abcam	ab113	1:400

*For 3D IHC, 5x the concentration of 2D antibodies was used in this study. Notably, this concentration might have to be adjusted dependent on antibody efficiency, availability of antigen and tissue size.

Supplementary Table S3 – Secondary Antibodies

Species	Anti-	Fluorophore	Producer	Cat#
**Donkey**	Mouse	AF488	Invitrogen	A21202
**Donkey**	Mouse	AF568	Invitrogen	A10037
**Donkey**	Mouse	AF647	Invitrogen	A31571
**Donkey**	Rabbit	AF488	Invitrogen	A21206
**Donkey**	Rabbit	AF568	Invitrogen	A10042
**Donkey**	Rabbit	AF647	Invitrogen	A31573
**Donkey**	Rat	AF488	Invitrogen	A21208
**Donkey**	Rat	AF647	Jackson ImmunoResearch	712-605-150
**Donkey**	Goat	AF488	Invitrogen	A11055
**Donkey**	Goat	AF568	Invitrogen	A11057
**Donkey**	Goat	AF647	Invitrogen	A21447
**Donkey**	Sheep	AF488	Invitrogen	A11015
**Donkey**	Sheep	AF568	Invitrogen	A21099
**Donkey**	Sheep	AF647	Jackson ImmunoResearch	713-605-147
**Donkey**	Chicken	AF488	Jackson ImmunoResearch	703-545-155
**Donkey**	Chicken	AF647	Jackson ImmunoResearch	703-605-155

Secondary antibodies were used 1:500 in 2D IHC and 1:100 in 3D IHC.
