# Supplementary material for: In vitro modeling of the human dopaminergic system using spatially arranged ventral midbrain–striatum–cortex assembloids
Source: Nat Methods. 2023 Dec 5;20(12):2034–47. doi: 10.1038/s41592-023-02080-x (PMC10703680; doi:10.1038/s41592-023-02080-x)
Supplement: Supplementary file 2 — Reporting Summary [file 41592_2023_2080_MOESM2_ESM.pdf]

nature portfolio

Corresponding author(s): Prof. Dr. Jürgen A. Knoblich

Last updated by author(s): 20.09.2023

Reporting Summary

Nature Portfolio wishes to improve the reproducibility of the work that we publish. This form provides structure for consistency and transparency in reporting. For further information on Nature Portfolio policies, see our Editorial Policies and the Editorial Policy Checklist.

Statistics

For all statistical analyses, confirm that the following items are present in the figure legend, table legend, main text, or Methods section.

n/a	Confirmed	
☐	☒	The exact sample size (*n*) for each experimental group/condition, given as a discrete number and unit of measurement
☐	☒	A statement on whether measurements were taken from distinct samples or whether the same sample was measured repeatedly
☐	☒	The statistical test(s) used AND whether they are one- or two-sided<br>*Only common tests should be described solely by name; describe more complex techniques in the Methods section.*
☐	☒	A description of all covariates tested
☐	☒	A description of any assumptions or corrections, such as tests of normality and adjustment for multiple comparisons
☐	☒	A full description of the statistical parameters including central tendency (e.g. means) or other basic estimates (e.g. regression coefficient) AND variation (e.g. standard deviation) or associated estimates of uncertainty (e.g. confidence intervals)
☐	☒	For null hypothesis testing, the test statistic (e.g. *F*, *t*, *r*) with confidence intervals, effect sizes, degrees of freedom and *P* value noted<br>*Give P values as exact values whenever suitable.*
☒	☐	For Bayesian analysis, information on the choice of priors and Markov chain Monte Carlo settings
☒	☐	For hierarchical and complex designs, identification of the appropriate level for tests and full reporting of outcomes
☒	☐	Estimates of effect sizes (e.g. Cohen's *d*, Pearson's *r*), indicating how they were calculated

Our web collection on statistics for biologists contains articles on many of the points above.

Software and code

Policy information about availability of computer code

Data collection

Commercial imaging software associated with Zeiss (Zen Blue), Olympus IX3 series and Visiscope spinning disk system was used.
For Silicon Probe recordings, the software Open Ephys GUI.

For Bulk RNAseq, STAR 2.7.7a and DESeq2 v1.36.0 as well as Voxhunt v1.0.1 were used.

Exact usage of data collection can be found in the Methods section.

PDMS molds were designed in Tinkercad (www.Tinkercad.com) and sliced using the slicer software XYZ print 1.4.0.

Data analysis

Images were processed using the FIJI distribution of the open-source image processing application ImageJ (version 1.53q).

GCAMP trace extraction was performed using the open-source software package CaImAn (Flatiron Institute). Spike detection and event duration were performed using a custom code in Python 3.9.

Statistical analysis was performed using GraphPad Prism 9.4.1.

(sc)RNAseq analysis was performed using R Studio (2022.07.0 Build 548 and newer) and Cellranger count v7.0.0 and Seurat v4.1.1 were used.

Details of exact usage as well as data availability is provided in the Methods section.

Code used for analysis of data in this study have been deposited in a Github repository and is publicly available (https://github.com/bardylab/miscos_org_ephys).

For manuscripts utilizing custom algorithms or software that are central to the research but not yet described in published literature, software must be made available to editors and reviewers. We strongly encourage code deposition in a community repository (e.g. GitHub). See the Nature Portfolio guidelines for submitting code & software for further information.

Data

Policy information about availability of data

All manuscripts must include a data availability statement. This statement should provide the following information, where applicable:

- Accession codes, unique identifiers, or web links for publicly available datasets
- A description of any restrictions on data availability
- For clinical datasets or third party data, please ensure that the statement adheres to our policy

Bulk and single cell RNA-seq data generated in this study have been made available at NCBI Gene Expression Omnibus (GEO) under the accession number GSE219247. Data and other unique reagents or biological materials generated in this study are available from the lead contact Dr. Juergen Knoblich (juergen.knoblich@imba.oeaw.ac.at) upon reasonable request and in compliance with Material Transfer Agreements (MTA). A STL CAD file for the embedding mold negative will be provided in the supplements.

Human research participants

Policy information about studies involving human research participants and Sex and Gender in Research.

Reporting on sex and gender	n/a
Population characteristics	n/a
Recruitment	n/a
Ethics oversight	n/a

Note that full information on the approval of the study protocol must also be provided in the manuscript.

Field-specific reporting

Please select the one below that is the best fit for your research. If you are not sure, read the appropriate sections before making your selection.

☒ Life sciences ☐ Behavioural & social sciences ☐ Ecological, evolutionary & environmental sciences

For a reference copy of the document with all sections, see nature.com/documents/nr-reporting-summary-flat.pdf

Life sciences study design

All studies must disclose on these points even when the disclosure is negative.

Sample size	No predetermined sample size calculations were performed and sample size was determined by common practice in the field and based on previous studies in the field (Lancaster et al., Nature 2013, Lancaster et al., Nature Biotechnology 2017, Bagley et al., Nature Methods 2017, Esk&Lindenhofer et al., Science 2020, Eichmüller et al., Science 2022)
Data exclusions	No data which met the quality criteria of the designed experiments (e.g. read count in RNAseq) was excluded in this study.
Replication	All key experiments were successfully replicated at least twice and number of replicates are mentioned in figure legends.
Randomization	Organoids were randomly selected from organoid batches for each type of experiment.
Blinding	This study involved unbiased analysis and quantification for immunostaining, functional activity and gene expression data sets. Analysis was, where possible, performed on raw data by inherently unbiased pipelines. There was no expected outcome prior to any analysis, thus blinding was not performed.

Reporting for specific materials, systems and methods

We require information from authors about some types of materials, experimental systems and methods used in many studies. Here, indicate whether each material, system or method listed is relevant to your study. If you are not sure if a list item applies to your research, read the appropriate section before selecting a response.

Materials & experimental systems

n/a	Involved in the study	
☐	☒	Antibodies
☐	☒	Eukaryotic cell lines
☒	☐	Palaeontology and archaeology
☒	☐	Animals and other organisms
☒	☐	Clinical data
☒	☐	Dual use research of concern

Methods

n/a	Involved in the study	
☒	☐	ChIP-seq
☒	☐	Flow cytometry
☒	☐	MRI-based neuroimaging

Antibodies

Antibodies used

Primary Antibodies:
Species Antigen Producer Cat# Dilution used in 2D*
Mouse ALDH1A1 (Clone B-5) Santa Cruz Biotech sc-374149 1:50
Rabbit Calbindin Aves lab/Swant CD38a D-28K 1:1000
Rat CTIP2/ BCL11b Abcam ab18465 1:300
Rabbit DARPP32 (Clone EP720Y) Abcam ab40801 1:100
Goat DARPP32 R&D systems AF6259 1:100
Rat DAT (Clone DAT-NT) Millipore MAB369 1:1000
Goat DLX2 Santa Cruz Biotech sc-18140 1:100
Sheep DLX5 R&D Systems AF6710 1:100
Rabbit DRD1 Abcam ab20066 1:400
Rabbit DRD2 Millipore AB5084P 1:100
Rabbit DsRed/tdtomato/Crimson Clontech 632496 1:250
Rabbit EN2 LSBio LS-B9057-200 1:100
Goat FOXA2 R&D Systems AF2400 1:300
Rabbit FoxG1 Abcam ab18259 1:200
Rabbit FOXP1 Abcam AB16645 1:200
Rabbit GABA Sigma-Aldrich a2052 1:200
Mouse GAD67 (Clone 1G10.2) Millipore MAB5406 1:200
Chicken GFP Aves Labs GFP-1020 1:500
Rabbit GIRK2 (Kir3.2) Alomone Labs APC-006 1:400
Rabbit GSX2/GSH Millipore ABN162 1:100
Rabbit ISL-1 Abcam AB20670 1:100
Rabbit LMX1a Sigma-Aldrich AB10533 1:100
Rabbit MASH1/ASCL1 Abcam AB74065 1:100
Rabbit Nkx2.1/Thyroid (TTF1)
(Clone EPR5955(2)) Epitomics 6594-1 1:1000
Goat OTX2 R&D Systems AF1979 1:100
Mouse PAX6 (Clone AD2.38) Abcam ab78545 1:100
Sheep PAX6 R&D Systems AF8150 1:200 of 100 µl reconstitute
Rabbit RFP/tdtomato/Crimson Abcam ab62341 1:100
Rabbit SOX2 Abcam ab97959 1:600
Goat SOX2 R&D Systems AF2018 1:100
Rabbit SOX6 Abcam ab30455 1:500
Rabbit TBR1 Abcam ab31940 1:300
Rabbit TH Abcam ab112 1:300
Sheep TH Abcam ab113 1:400

Secondary Antibodies:
Species Anti- Fluorophore Producer Cat#
Donkey Mouse AF488 Invitrogen A21202
Donkey Mouse AF568 Invitrogen A10037
Donkey Mouse AF647 Invitrogen A31571
Donkey Rabbit AF488 Invitrogen A21206
Donkey Rabbit AF568 Invitrogen A10042
Donkey Rabbit AF647 Invitrogen A31573
Donkey Rat AF488 Invitrogen A21208
Donkey Rat AF647 Jackson ImmunoResearch 712-605-150
Donkey Goat AF488 Invitrogen A11055
Donkey Goat AF568 Invitrogen A11057
Donkey Goat AF647 Invitrogen A21447
Donkey Sheep AF488 Invitrogen A11015
Donkey Sheep AF568 Invitrogen A21099
Donkey Sheep AF647 Jackson ImmunoResearch 713-605-147

Donkey Chicken AF488 Jackson ImmunoResearch 703-545-155
Donkey Chicken AF647 Jackson ImmunoResearch 703-605-155

Validation

Mouse ALDH1A1 (Clone B-5) Santa Cruz Biotech sc-374149: Validated by the company and used in 26 scientific literatures.
Rabbit Calbindin Aves lab/Swant CD38a D-28K: Validated by the company and used in one scientific literature.
Rat CTIP2/ BCL11b Abcam ab18465: Validated by the company and used in 718 scientific literatures.
Rabbit DARPP32 (Clone EP720Y) Abcam ab40801: Validated by the company and used in 96 scientific literatures.
Goat DARPP32 R&D systems AF6259: Validated by the company and used in one scientific literature.
Rat DAT (Clone DAT-NT) Millipore MAB369: Validated by the company.
Goat DLX2 Santa Cruz Biotech sc-18140: Validated by the company and used in 6 scientific literatures.
Sheep DLX5 R&D Systems AF6710: Validated by the company.
Rabbit DRD1 Abcam ab20066: Validated by the company and used in 47 scientific literatures.
Rabbit DRD2 Millipore AB5084P: Validated by the company.
Rabbit DsRed/tdtomato/Crimson Clontech 632496: Validated by the company and used in 2004 scientific literatures.
Rabbit EN2 LSBio LS-B9057-200: Validated by the company and used in one scientific literature.
Goat FOXA2 R&D Systems AF2400: Validated by the company and used in 66 scientific literatures.
Rabbit FoxG1 Abcam ab18259: Validated by the company and used in 126 scientific literatures.
Rabbit FOXP1 Abcam AB16645: Validated by the company and used in 97 scientific literatures.
Rabbit GABA Sigma-Aldrich a2052: Validated by the company.
Mouse GAD67 (Clone 1G10.2) Millipore MAB5406: Validated by the company.
Chicken GFP Aves Labs GFP-1020: Validated by the company and used in 747 scientific literatures.
Rabbit GIRK2 (Kir3.2) Alomone Labs APC-006: Validated by the company and used in 187 scientific literatures.
Rabbit GSX2/GSH Millipore ABN162: Validated by the company.
Rabbit ISL-1 Abcam AB20670: Validated by the company and used in 83 scientific literatures.
Rabbit LMX1a Sigma-Aldrich AB10533: Validated by the company.
Rabbit MASH1/ASCL1 Abcam AB74065: Validated by the company and used in 36 scientific literatures.
Rabbit Nkx2.1/Thyroid (TTF1) (Clone EPR5955(2)) Epitomics 6594-1: Validated by the company and used in 2 scientific literatures.
Goat OTX2 R&D Systems AF1979: Validated by the company and used in 81 scientific literatures.
Mouse PAX6 (Clone AD2.38) Abcam ab78545: Validated by the company and used in 44 scientific literatures.
Sheep PAX6 R&D Systems AF8150: Validated by the company and used in 10 scientific literatures.
Rabbit RFP/tdtomato/Crimson Abcam ab62341: Validated by the company and used in 290 scientific literatures.
Rabbit SOX2 Abcam ab97959: Validated by the company and used in 656 scientific literatures.
Goat SOX2 R&D Systems AF2018: Validated by the company and used in 196 scientific literatures.
Rabbit SOX6 Abcam ab30455: Validated by the company and used in 56 scientific literatures.
Rabbit TBR1 Abcam ab31940: Validated by the company and used in 425 scientific literatures.
Rabbit TH Abcam ab112: Validated by the company and used in 332 scientific literatures.
Sheep TH Abcam ab113: Validated by the company and used in 63 scientific literatures.

Eukaryotic cell lines

Policy information about cell lines and Sex and Gender in Research

Cell line source(s)

The hESC lines (H1 (WAe001-A), H7 (WA07), H9 (WA09) (all WiCell) and in-House (IMBA Stem Cell Core Facility) generated human iPSC lines (176/1, 178/5, 178/6) were used in this study.

Authentication

Cell lines were authenticated by WiCell or IMBA Stem Cell Core Facility and checked for pluripotency and genomic integrity. Cell lines were checked to be morphologically consistent and show no signs of differentiation before every passage.

The protocol and informed consent form regarding iPSC derivation for the cell lines SCCF-176J clone#1, SCCF-178 clone#5 and #6 was reviewed and approved by the properly constituted Institutional Review Board/Independent Ethics Committee at the Medical University of Vienna (EK No. 1596/2017).

Cell lines SCCF-176J clone#1 and SCCF-178 clone#5 have been whole-genome sequenced and sequences are available from the EGA database (Study ID EGAS00001006262; Dataset: https://ega-archive.org/datasets/EGAD00001008769). Cell lines SCCF-176J clone#1 (https://hpscreg.eu/cell-line/IMBAi001-A), SCCF-178 clone#5 (https://hpscreg.eu/cell-line/IMBAi003-A) and SCCF-178 clone#6 (https://hpscreg.eu/cell-line/IMBAi003-B) have been registered at the Human Pluripotent Stem Cell Registry (https://hpscreg.eu/).

Mycoplasma contamination

All cell lines were routinely tested to be negative for mycoplasma contamination.

Commonly misidentified lines
(See ICLAC register)

No common misidentified lines have been used in this study.
